# In The Line of Treatment: A Systematic Review of Paroxysmal Supraventricular Tachycardia

**DOI:** 10.7759/cureus.15502

**Published:** 2021-06-07

**Authors:** Farrukh Ahmad, Majdi Abu Sneineh, Ravi S Patel, Sai Rohit Reddy, Adiona Llukmani, Ayat Hashim, Dana R Haddad, Domonick K Gordon

**Affiliations:** 1 Emergency Medicine, California Institute of Behavioral Neurosciences & Psychology, Fairfield, USA; 2 Emergency Medicine, Beaumont Hospital, Dublin, IRL; 3 Internal Medicine, California Institute of Behavioral Neurosciences & Psychology, Fairfield, USA; 4 Medical Education and Simulation, California Institute of Behavioral Neurosciences & Psychology, Fairfield, USA; 5 Internal Medicine/Pediatrics, California Institute of Behavioral Neurosciences & Psychology, Fairfield, USA; 6 Plastic and Reconstructive Surgery, California Institute of Behavioral Neurosciences & Psychology, Fairfield, USA

**Keywords:** calcium channel blockers, adenosine, supraventricular tachycardia

## Abstract

Paroxysmal* *supraventricular tachycardia (PSVT) is a common tachyarrhythmia, and an electrocardiogram is the best tool for making a diagnosis. If Valsalva maneuvers and carotid sinus massage do not give positive results, then the next choice is either adenosine or calcium channel blockers. At this time, adenosine is the drug of choice of treatment. Verapamil and diltiazem are the most commonly used calcium channel blockers (CCBs). This review aimed to compare the efficacy of both drugs in the treatment of PSVT.

We utilized the databases PubMed Central and Medline by using keywords: "calcium channel blockers OR adenosine AND supraventricular tachycardia." In the end, we finalized 32 studies, including observational studies, literature reviews, systematic reviews/metanalysis, and randomized control trials. We included articles only in the English language and related to humans. Two authors completed the quality assessment and evaluation of bias according to specific guidelines. Only high-quality studies were included in this systematic review based on the cut-off score of seven or above. Calcium channel blockers have a longer half-life than adenosine and were previously used as the drug of choice in the treatment of PSVT. Calcium channel blockers are safe if given slowly; however, adenosine is safer and useful when an electrocardiogram is uncertain. We compared both drugs in certain aspects and found equal efficacy. Though safer, adenosine was found to have a higher cost and a higher probability of re-initiation arrhythmia compared to calcium channel blockers.

## Introduction and background

According to the Heart Rhythm Society, millions of individuals encounter unusual heartbeats at some stage in their lives. Most of the time, they are safe and happen in individuals free of heart illnesses. However, a few abnormal heart rhythms can be genuine or indeed dangerous. Having underlying heart disease can also increase the chance of arrhythmias [1].

**Figure 1:**
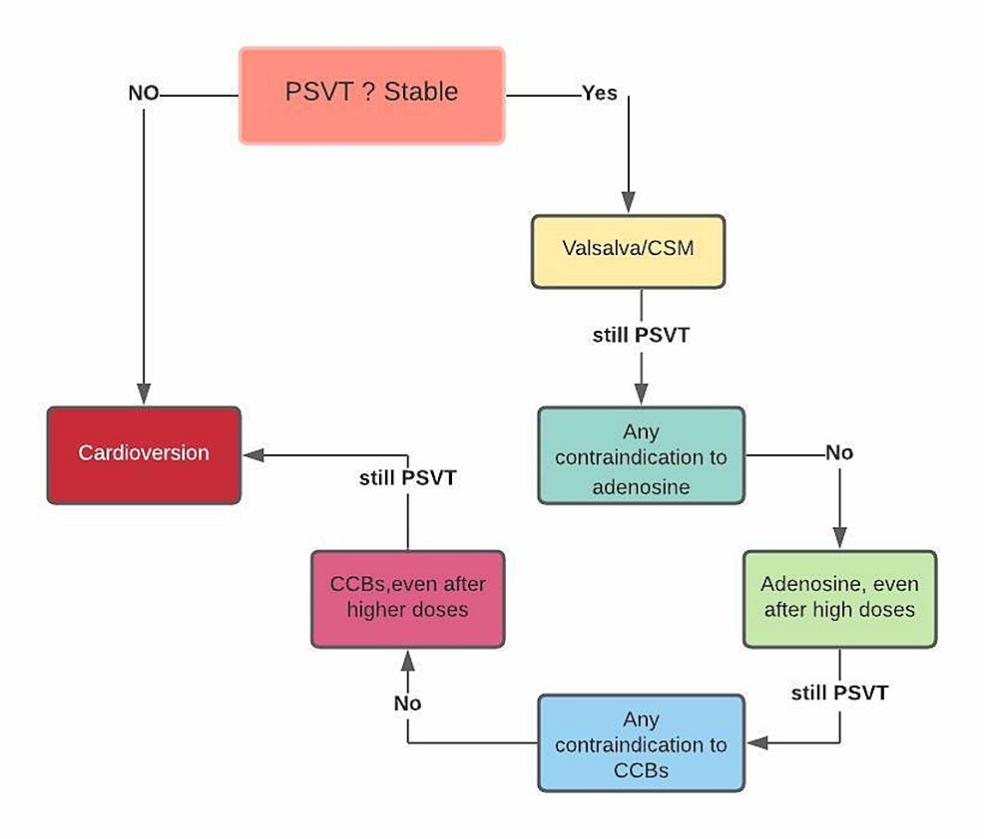
Treatment Options for Paroxysmal Supraventricular Tachycardia PSVT= paroxysmal supraventricular tachycardia, CCBs= calcium channel blockers, CSM= carotid sinus massage

Paroxysmal supraventricular tachycardia (PSVT) alludes to fast rhythms that start and is sustained in atrial or atrioventricular hub tissue over the bundle of His. PSVT is caused by re-entry phenomena or automaticity at or over the atrioventricular node. PSVT includes atrioventricular nodal re-entrant tachycardia (AVNRT), atrioventricular reciprocating tachycardia (AVRT), atrial tachycardia (AT), and a few more tachyarrhythmias [2]. Accelerated rhythms can be terrifying to the patient and can cause significant morbidity. AVNRT is the most common type of paroxysmal SVT, followed by AVRT [3]. For patients presenting with PSVT, a 12-lead electrocardiogram (ECG) showing a narrow complex tachycardia is the basis for making the diagnosis and uncovering the arrhythmia mechanism [4]. In pregnancy, the most common tachyarrhythmia is AVNRT [5]. There are a few treatment choices for PSVT, as seen in Figure 1.

The most commonly performed initial treatment is the Valsalva maneuver and carotid sinus massage (CSM). The increase in intra-thoracic pressure from these maneuvers can stimulate aortic and carotid baroreceptors, causing an increased firing of vagal input into the atrioventricular hub [6]. Failure of CSM could be due to inadequately performed massage and a decrease in the response of PSVT over time [7]. When these specific maneuvers are unsuccessful, PSVT can be treated within the emergency department with an assortment of various drugs. PSVT is more common in women than men, with an average age of around 55 years [8]. 

Adenosine is an endogenous nucleoside with a half-life of less than a minute, which acts by repressing calcium influx and improving potassium conduction. Adenosine leads to inhibition of atrioventricular (AV) nodal conduction and increases the AV nodal refractory period. Due to its brief half-life, reversion to sinus beat may be short-lived as an ensuing ectopic beat may re-initiate SVT. Numerous patients encounter short-lived but exceptionally unpleasant side impacts after adenosine administration, such as dyspnoea, flushing, and a sense of impending doom that may be incredibly frightening [9]. Adenosine though more expensive than other intravenous medications is still the drug of choice [10].

Calcium channel blockers (CCBs) like verapamil inhibit calcium ion influx in direct proportion to its concentration in plasma. It has a bioavailability of 20-35%, has no or few active metabolites, eliminated by extra-renal routes, and has a plasma half-life of three to six hours. It reaches its peak plasma level in one to two hours [11]. CCBs can cause negative inotropy and peripheral vasodilation resulting in hypotension, particularly in patients with impaired left ventricular function [9]. If there is a hemodynamic compromise in patients with PSVT, then the best option is direct current cardioversion [12].

This review aims to compare different aspects of both drugs, such as reversion rate to normal sinus rhythm, time to immediate reversion to sinus rhythm, cost of medicine, minor and major side effects, recurrence of arrhythmia, and the better option. We also aim to determine if oral CCB is a suitable alternative after the failed termination of PSVT with adenosine.

## Review

This systematic review compared calcium channel blockers versus adenosine in the treatment of supraventricular tachycardia. We followed the Preferred Reporting Items for Systematic Reviews and Meta-Analyses (PRISMA) guidelines. 

Methods

**Figure 2:**
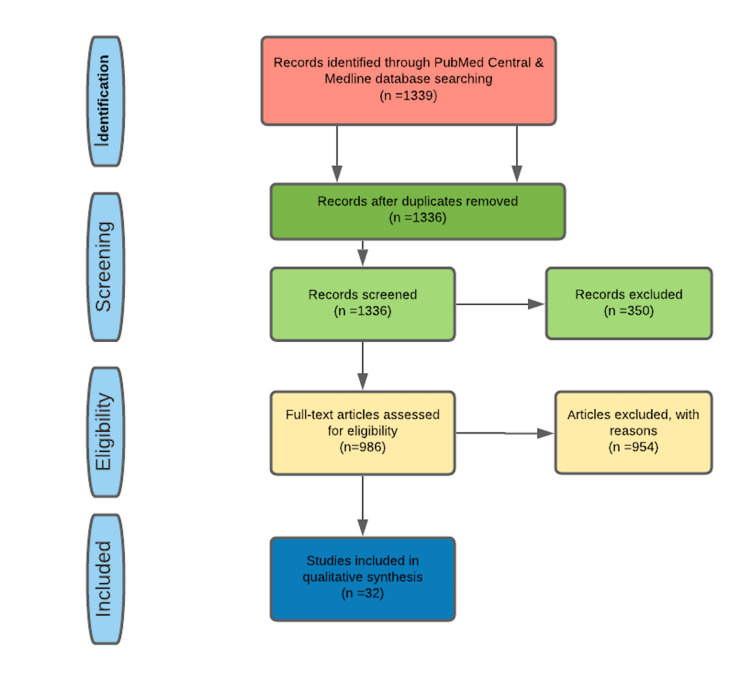
Search Strategy PRISMA Flow Diagram PRISMA= Preferred Reporting Items for Systematic Reviews and Meta-Analyses

The search included the electronic databases Medline and PubMed Central. The keywords used in the search process included "calcium channel blockers OR adenosine AND supraventricular tachycardia" and ("calcium channel blockers" [MeSH major topic] OR "adenosine" [MeSH major topic]) AND "tachycardia, supraventricular" [MeSH major topic], medical subject headings (MeSH) strategy, used. There were 1339 articles identified from November 1972 to December 26, 2020. After applying our inclusion/exclusions criteria, which consisted of only humans and the English language, the number of articles reduced to 989. Mendeley citation manager was used to remove duplicated studies. In the end, 954 studies were excluded on the basis of title and abstract and 32 were retained. Figure 2 below shows the search strategy PRISMA flow diagram.

Quality of Study

According to each specific guideline, two authors checked each study's quality in the review without blinding to authorship or journal for the risk of bias. We found all studies of high quality according to the quality assessment tools.

Types of Studies and Outcomes Measured

The final 32 studies contain randomized clinical trials (RCT), observational studies, non-randomized clinical trials, case reports, traditional reviews, editorial letters, and systematic review/metanalysis.

The outcome measures that were used to compare CCBs and adenosine in the treatment of PSVT were (1) rate of reversion to normal sinus rhythm, (2) time to immediate reversion to sinus rhythm, (3) cost of medicine, (4) minor and major side effects, and (5) recurrence of arrhythmia.

Results

The search identified 1339 potentially relevant studies on PubMed Central and Medline. The majority of studies were excluded on the basis of the relevance of the abstract and title. We used the Mendeley reference manager to remove duplicate studies. No research before 1972 was included. The summary of the final 32 studies shown below in Table 1.

**Table 1:** Summary of Included Studies AD= adenosine, CCB= calcium channel blockers, RCT= randomiazed control trials, SR= systematic review, SVT= supraventricular tachycardia, PSVT= paroxysmal SVT, COPD= chronic obstructive pulmonary disease, ECG= electrocardiogram, VM= verapamil, DM= diltiazem, IV=intravenous

Article no.	Author	Year of publication	No. of participants	Aim of study	Quality assessment	Assessment score	Type of study	Findings
1	Schamroth et al. [13]	1972	181	Immediate effect of IV verapamil in cardiac arrhythmias	High quality	7	Observational study	Verapamil for the immediate control of a variety of cardiac arrhythmias has been excellent
2	Krikler and Spurrell [14]	1974	32	Verapamil in the treatment of PSVT	High quality	8	SR/meta-analysis	Verapamil has been shown to be a safe and effective agent for SVT
3	Vohra et al. [15]	1974	4	Cycle length alteration in SVT after administration of verapamil	High quality	16	Case report	Verapamil given by IV route was of value in the treatment of SVT
4	Wellens et al. [16]	1977	10	Effect of verapamil studied by program-med electric-al stimulation of the heart in patients with SVT	High quality	8	Observational study	Verapamil resulted in a slowing of the heart rate during tachycardia
5	Rabkin et al. [17]	1980	11	CCB and SVT with COPD	High quality	7	Observational study	CCB is effective in SVT with COPD
6	Kenny [18]	1985	Nil	CCBs and the heart	High quality	7	Editorial letter	CCB is safe and effective in Heart diseases
7	Krikler [19]	1986	Nil	Verapamil in arrhythmia	High quality	11	Traditional review	Verapamil is highly effective
8	Sternbach et al. [20]	1986	11	IV diltiazem for the treatment of SVT	High quality	9	Traditional review	Diltiazem is safe and effective
9	Gutman [21]	1987	Nil	Selecting a CCB	High quality	8	Traditional review	CCB was good in SVT
10	Ornato et al. [7]	1988	16	Treatm-ent of PSVT in ED	High quality	7	Traditional review	CCB was safe in old age
11	DiMarco et al. [22]	1990	357	AD for PSVT and comparison with VM	High quality	7	RCT	Both AD and CCB were equal in efficacy
12	Byerly et al. [23]	1991	2	Verapa-mil in treatm-ent of matern-al PSVT	High quality	10	Case report	CCB was safe in pregnant patients
13	Hood and Smith [24]	1992	25	AD vs. verapamil in the treatment of SVT	High quality	7	RCT	AD was better than CCB in the treatment of SVT
14	Dougherty et al. [25]	1992	87	Acute conversion of PSVT with iv diltiazem	High quality	7	RCT	Diltiazem is effective in PSVT
15	Peitz [26]	1993	Nil	IV diltiazem rather than verapamil in PSVT	High quality	8	Editorial letter	Diltiazem was effective in PSVT
16	Madsen et al. [27]	1995	191	AD and verapamil for SVT in the prehospital setting	High quality	7	Observational study	Verapamil and AD are equal in efficacy
17	Brady et al. [28]	1996	211	Treatment of out of hospital SVT AD vs. verapamil	High quality	8	Observational study	Both AD and verapamil were equal in efficacy
18	Ou et al. [29]	2004	1	Choosing CCB for pregnant women with PSVT	High quality	14	Case report	CCB is safe in pregnant patients
19	Holdgate and Foo [10]	2006	577	AD vs. CCB for treatment of SVT in adults	High quality	12	SR/meta-analysis	Both AD and CCB were equal in efficacy
20	Anugwo et al. [30]	2007	Nil	AD vs. CCB for SVT	High quality	7	Editorial letter	AD, the first line of drug
21	Lim et al. [31]	2009	233	Slow iv CCB vs. iv AD in treatment of SVT	High quality	8	RCT	AD, the first line of drug
22	Turkoglu et al. [32]	2009	74	verapamil and AD in termination of sustain-ed SVT	High quality	8	RCT	CCB was found highly effective
23	Colucci et al. [3]	2010	Nil	Common types of SVT: diagnosis and management	High quality	8	Traditional review	Ablation is overall better for the treatment of SVT
24	Ghosh et al. [33]	2011	Nil	Acute treatment of maternal SVT in pregnancy	High quality	10	Traditional review	AD was a safe choice in pregnancy
25	Smith et al. [8]	2014	933	Prehospital management of SVT in Victoria Australia	High quality	7	Observational study	Underutilisation of therapies
26	Sohinki and Obel [6]	2014	Nil	Current trends in SVT management	High quality	9	Traditional review	Ablation, overall better to treat SVT
27	Dogan et al. [34]	2015	77	AD or diltiazem in SVT in Emergency Dept.	High quality	7	Observational study	Diltiazem was a better option than AD
28	Helton [2]	2015	Nil	Diagnosis and management of common types of SVT	High quality	8	Traditional review	Ablation is an overall better option to treat SVT
29	Shaker et al. [11]	2015	92	Oral ver-apamil in PSVT recurre-nce control	High quality	9	RCT	Use of CCB after AD in SVT was recommended
30	Alabed et al. [4]	2017	622	AD vs CCB for SVT	High quality	10	SR/meta-analysis	Both AD and CCB were equal in efficacy
31	Brubaker et al. [5]	2018	Nil	Alterna-te treatm-ent option for PSVT	High quality	8	Traditional review	Both AD and CCB were equal in efficacy
32	Kotodia et al. [12]	2020	Nil	SVT: An overview of diagnosis and management	High quality	7	Traditional review	ECG has a key role in the long-term treatment of SVT

The outcome was 32 studies included, 350 studies removed after applying inclusion and exclusion criteria, and 954 studies removed based on the abstract and title. The final 32 studies consisted of systematic review/metanalysis, literature reviews, observational studies, editorial letters, randomized controlled trials, and case report/case series. We included studies done in the hospitals and few studies showing data related to prehospital settings, with a population of adults and children. The included case report/case series focused on pregnant patients. The total number of patients was 3111 in the final 32 studies. Studies were ranked high quality based on the score of quality assessment tools, and the cut-off score was equal to or more than seven.

The research question was "are calcium channel blockers a better choice than adenosine in supraventricular tachycardia?"

Inclusion and Exclusion Criteria

The human studies only in the English language were included and the studies before 1972 and animal studies were excluded.

Bias risk in included studies: Through quality assessment tools, two authors evaluated the risk of selection bias by assessing randomization and allocation concealment. They ranked performance, detection, and attrition bias by assessing blinding to treatment, blinding to outcome assessment, and converting each study to high risk, low risk, and unclear risk.

Discussion

The purpose of this review is to compare the safety and efficacy of CCBs with adenosine in patients presenting with PSVT. Studies in this article include prehospital, hospital-based patients from childhood to adulthood and pregnant patients experiencing PSVT. This article focuses on the following five points: (1) rate of reversion to normal sinus rhythm, (2) time to immediate reversion to sinus rhythm, (3) minor and significant side effects, (4) recurrence of arrhythmia, and (5) cost of medicine.

The most commonly found type of PSVT in the general population is AVRNRT [35]. In AVRNT, P-waves are challenging to see on the electrocardiogram strip (ECG) due to more or less the same time activation of both atria and ventricle [11]. Either P-waves present as a pseudo-R-wave in lead V1 or a pseudo-S' deflection in inferior leads, but P-waves can be subdued. Pseudo-R-wave is a more critical ECG finding and having high sensitivity regarding diagnosis [36]. Another finding on ECG is an aVL notch, which is any positive deflection at the end of the Q-wave, R-wave, and S-wave (QRS) complex during tachycardia but absent in normal sinus rhythm [34]. 

Adenosine

Adenosine has been established as the first-line drug treatment of PSVT due to its comparatively short half-life and safe drug profile. Most clinicians are now using it not only for a therapeutic purpose but also for diagnostic purposes [26]. Türkolu et al. found the appearance of ventricular complexes during the termination of AVNRT, which was more related to adenosine than CCBs [32]. The recommended dosage of adenosine and calcium channel blockers is shown below in Table 2.

**Table 2:** Showing the Dosing for Adenosine and Calcium Channel Blockers.

Drug	Initial intravenous dose	Further dosing if unsuccessful
Diltiazem	0.25 mg/kg over 2 mins	Further 0.35 mg/kg after 15 mins
Verapamil	5-10 mg over 5 mins	Further 5-10 mg after 5 mins
Adenosine	6 mg stat	Further 12 mg after 1-2 mins

Studies have shown that adenosine prevents sinus node automaticity, suppresses atrioventricular node conduction, refractoriness, and some drug-induced ventricular arrhythmias [22-37].

Calcium channel blockers

In cardiac and smooth muscle, CCBs block calcium movements across the cell membrane. CCBs prevent smooth muscle contraction during the depolarization phase, leading to decreased cardiac muscle tone and myocardial contractility [38]. Verapamil and diltiazem are the most commonly used CCBs in the treatment of PSVT. CCBs are contraindicated in patients with atrioventricular blocks, except for first-degree, sick sinus syndrome and digoxin toxicity [21]. Oral verapamil has shown a good response for prophylaxis of AVNRT [19]. Due to its quick action, Wellens et al. used intravenous (IV) verapamil as the first drug of choice for AVNRT after the failure of vagal maneuvers [16]. Krikler and Spurrell documented the rapid conversion of PSVT into sinus rhythm with IV verapamil [14]. Anugwom et al. recommended the use of CCBs if PSVT came back with the initial use of adenosine [30]. CCBs are also known to be beneficial in treating pulmonary hypertension and hypertrophic cardiomyopathy [18]. Dougherty et al. found that hypotension was the most common side effect of diltiazem [25]. Sternbach et al. also showed that intravenous (IV) diltiazem can be as effective and a safe alternative to IV verapamil for AVNRT [20]. 

Adenosine vs. calcium channel blockers

In one study, two of 11 identified patients had PSVT and were treated with verapamil. With a background history of ischemic cardiac disease, one patient who suffered from a transient atrioventricular block had to be installed with a radiofrequency pacemaker and coronary sinus catheter [13]. In a case report, four patients received IV verapamil, and all converted to sinus rhythm; however, the review was to see morphological changes in ECG after verapamil administration [15].

Two prospective, double-blind, randomized, placebo-controlled trials showed that sequential adenosine injections (6 mg and 12 mg) produced the same highly effective verapamil results with 5 mg and 7.5 mg. However, one-third of patients suffered from a few adverse reactions but of brief duration. The conversion rate was quick with both drugs, but re-initiation of arrhythmia was more common with adenosine use [17].

A total of 25 of 32 patients received adenosine and verapamil. Adenosine was quick in producing results with brief side effects; however, re-initiation of arrhythmia occurred in two patients out of 14 in a randomized double-crossover trial. In contrast, three of 11 patients treated with verapamil did not convert to sinus rhythm due to failure to terminate tachycardia in two and hypotension in one patient. A total of 17 patients were treated with adenosine, but 13 experienced side effects of drug-like chest tightness, feelings of electric shock, and flushing with the conversion dose. The study supported adenosine as the first-line treatment for PSVT due to the drugs` short life of a few seconds, therefore having less myocardial depression than verapamil [24].

Verapamil showed 64% successful results in the prehospital setting during the verapamil period, converting SVT to sinus rhythm, while adenosine had 78% successful results. In this prehospital setting, paramedics received instructions from hospital-based physicians. The study found that the physicians were more interested in adenosine versus verapamil. SVT was defined as either AVNRT and AVRT with a heart rate between 160 and 240 with sudden onset. Verapamil caused 29% of side effects, including hypotension. On the other hand, adenosine caused 45% side effects, but none required emergency treatment. One limitation of this study was that paramedics and hospital-based physicians misdiagnosed 41% of SVT confirmed with cardiologists [27].

In another prehospital study, verapamil was given in 52 patients with SVT, 43 converted to sinus rhythm, but 17 of 43 had a recurrence of SVT, two in the prehospital setting and 15 in the emergency department [28]. Adenosine was given to 87 patients and 60 patients converted to sinus rhythm. Twenty-five of 60 patients showed SVT recurrence, two out of the hospital, and 23 in the hospital. In another study, drugs were given in different time durations and three out of four patients experienced minor side effects. One had prolonged bradycardia due to prior use of dipyridamole in the adenosine group. Two patients had ventricular tachycardia, ventricular fibrillation, and hypotension seen in one patient each. However, the conversion rate was high with verapamil in the first dose, but the second and third dose rate of conversion was high in adenosine. The study also found a higher adenosine price compared to verapamil [28].

In one prospective randomized controlled clinical trial, patients presented with SVT. Verapamil and diltiazem were used and compared with adenosine. Overall, adenosine showed an 86.5% conversion rate. In contrast, verapamil and diltiazem both had a 97.9% and 98.1% conversion rate, respectively. One patient experienced hypotension secondary to verapamil. Minor side effects were common with adenosine, as seen in 76% of patients. SVT recurrence was seen in two patients with adenosine, one with diltiazem, and none in verapamil in post-drug two hours. Overall treatment cost was high in adenosine followed by diltiazem and verapamil [31].

In another study, 92 patients were recruited in a randomized clinical trial. One group had adenosine only and the other group had adenosine/verapamil. Both groups were observed for two hours after treatment. The results showed 45% recurrence in the adenosine group and 28% recurrence in the adenosine/verapamil group. Two patients experienced minor side effects in the adenosine group, and one patient in the adenosine/verapamil group developed reduced systolic blood pressure [11]. 

Interpretation and analysis

Based on the data analyzed in Table 3, adenosine has a high conversion rate and quick mechanism of action in converting PSVT into sinus rhythm than CCBs; however, it is also associated with a higher cost and a higher recurrence of arrhythmia. 

**Table 3:** Comparison of Studies AD= adenosine, CCBs= calcium channel blocker

Author name	Rate of reversion to normal sinus rhythm	Time to immediate reversion to sinus rhythm	Minor and major side effects	Recurrence of arrhythmia	Cost of medicine	Type of study
DiMarco et al. [22]	Both AD and CCB have an equal rate of reversion	Both AD and CCB quick in time to reversion	AD=more minor effects CCB=less minor effects+ major effects	AD was related to recurrence CCB showed no recurrence	no comment	Two prospective, double-blind, randomized, placebo-controlled trials
Hood and Smith et al. [24]	AD has more rate of reversion than CCB	AD was quicker than CCB	AD=minor effects	AD was related to recurrence CCB showed no recurrence	no comment	Randomized double-crossover trial
Madsen et al. [27]	AD has more rate of reversion than CCB	No comment	AD=more minor effects CCB=less	AD high recurrence CCB has less recurrence	AD=high cost CCB=less	12 months chart review of AD and CCBs administrations
Brady et al. [28]	AD=less in the first dose CCB=high in the first dose	AD was quicker than CCB	AD=more minor effects CCB=less minor effect+ Major effects	AD=recurrence CCB=recurrence	AD=high cost CCB=less	A comparison of prospective AD use with prospective CCBs use
Lim et al. [31]	AD=less CCB=high	AD was quicker than CCB	AD=more minor effects CCB=less	AD=recurrence CCB=recurrence	AD=high cost CCB=less	Prospective randomized controlled clinical trial

Pregnancy and PSVT 

A case report documented the successful conversion of narrow complex, regular SVT without delta wave in a pregnant patient with verapamil. Still, it was unsuccessful with the same patient even a week later. The second time, a dropped maternal blood pressure was recorded; however, no ill effect occurred on the fetus on both occasions [23]. Madsen et al. reported an out-of-hospital conversion of SVT to sinus rhythm with adenosine in a thirty-week pregnant patient with no adverse effects seen [27]. Verapamil has also shown excellent results, lacking fetal and maternal side effects [39]. Ghosh et al. found adenosine more successful than CCBs and recommend using adenosine first. If not successful, especially in the second and third-trimester, beta-blockers should be used before verapamil, and both mother and fetus need to be monitored [33]. Ou et al. assumed that calcium channel blockers were preferable to conventional tocolytic agents in preterm labor cases with PSVT [29].

Limitations

Our reviews' significant limitations included that only studies in the English language were included. Articles available in other languages that were excluded may have additional information to improve this paper's quality.

## Conclusions

Adenosine is the recommended first-line drug treatment for paroxysmal supraventricular tachycardia (PSVT), a benign arrhythmia. Adenosine and calcium channel blockers (CCBs) showed promising results regarding safety and efficacy. Adenosine has a comparatively short half-life and quick mechanism of action; however, it is also associated with a higher drug cost, unpleasant side effects, and a higher recurrence of arrhythmia. 

Slow intravenous CCBs can convert PSVT into sinus rhythm, provided no limitations and contraindications to the use of CCBs. We found different suggestions about treatment options for PSVT. A timely review of the ECG can make a difference in the treatment of PSVT. This review found that both adenosine and CCBs are good options in PSVT treatment. But adenosine is having clear advantages over CCBs and first drug of choice in the treatment of PSVT. Our recommendation is, after the successful termination of PSVT with adenosine, oral CCBs can be given if patients are suitable candidates for CCBs. We strongly recommend for future researchers do the study on it. In this way, we can avoid hospital stays, further costs of medications, and re-initiating arrhythmia.
